# Evaluation of Trimethoprim/Sulfamethoxazole (SXT), Minocycline, Tigecycline, Moxifloxacin, and Ceftazidime Alone and in Combinations for SXT-Susceptible and SXT-Resistant *Stenotrophomonas maltophilia* by In Vitro Time-Kill Experiments

**DOI:** 10.1371/journal.pone.0152132

**Published:** 2016-03-21

**Authors:** Chuanqi Wei, Wentao Ni, Xuejiu Cai, Jin Zhao, Junchang Cui

**Affiliations:** 1 Department of Respiratory Diseases, Chinese People’s Liberation Army General Hospital, Beijing 100853, China; 2 Department of Respiratory Diseases, Guangzhou General Hospital of Guangzhou Army Command of Chinese People’s Liberation Army, Guangzhou 510010, Guangdong, China; NERC Centre for Ecology & Hydrology, UNITED KINGDOM

## Abstract

**Background:**

The optimal therapy for infections caused by *Stenotrophomonas maltophilia* (*S*. *maltophilia*) has not yet been established. The objective of our study was to evaluate the efficacy of trimethoprim/sulfamethoxazole (SXT), minocycline, tigecycline, moxifloxacin, levofloxacin, ticarcillin-clavulanate, polymyxin E, chloramphenicol, and ceftazidime against clinical isolated *S*. *maltophilia* strains by susceptibility testing and carried out time-kill experiments in potential antimicrobials.

**Methods:**

The agar dilution method was used to test susceptibility of nine candidate antimicrobials, and time-killing experiments were carried out to evaluate the efficacy of SXT, minocycline, tigecycline, moxifloxacin, levofloxacin, and ceftazidime both alone and in combinations at clinically relevant antimicrobial concentrations.

**Results:**

The susceptibility to SXT, minocycline, tigecycline, moxifloxacin, levofloxacin, ticarcillin-clavulanate, chloramphenicol, polymyxin E, and ceftazidime were 93.8%, 95.0%, 83.8%, 80.0%, 76.3%, 76.3%, 37.5%, 22.5%, and 20.0% against 80 clinical consecutively isolated strains, respectively. Minocycline and tigecycline showed consistent active against 22 SXT-resistant strains. However, resistance rates were high in the remaining antimicrobial agents against SXT-resistant strains. In time-kill experiments, there were no synergisms in most drug combinations in time-kill experiments. SXT plus moxifloxacin displayed synergism when strains with low moxifloxacin MICs. Moxifloxacin plus Minocycline and moxifloxacin plus tigecycline displayed synergism in few strains. No antagonisms were found in these combinations. Overall, compared with single drug, the drug combinations demonstrated lower bacterial concentrations. Some combinations showed bactericidal activity.

**Conclusions:**

In *S*. *maltophilia* infections, susceptibility testing suggests that minocycline and SXT may be considered first-line therapeutic choices while tigecycline, moxifloxacin, levofloxacin, and ticarcillin-clavulanate may serve as second-line choices. Ceftazidime, colistin, and chloramphenicol show poor active against *S*. *maltophilia*. However, monotherapy is inadequate in infection management, especially in case of immunocompromised patients. Combination therapy, especially SXT plus moxifloxacin, may benefit than monotherapy in inhibiting or killing *S*. *maltophilia*.

## Introduction

*S*. *maltophilia* has increasingly become an important opportunistic pathogen in hospital settings. Hospital-acquired pneumonia (HAP) is the most common such infection, most often affecting immunosuppressed or debilitated patients [1]. Results from the SENTRY Antimicrobial Surveillance Program showed that this bacterium being the 6^th^ and 9^th^ most frequent occurrence of organisms isolated from patients hospitalized with pneumonia (2009–2012) in the USA and Europe and the Mediterranean region [2]. The characteristic of colonization in a variety of inanimate or animated surfaces such as catheters, water taps, the hands, and the respiratory tract grants its potential threat in hospital settings [1]. Although considered to be of limited pathogenicity, this bacillus is associated with high rates of morbidity and mortality, especially among immunosuppressed or debilitated patients [3]. Due to its intrinsic resistance to a variety of antimicrobials, including most β-lactams, quinolones, aminoglycosides, and tetracyclines, therapeutic options are often limited [1,3]. Based on in vitro activity, the “drug of choice” has long been SXT [1,3]. However, adverse reactions from, and resistance to, this antimicrobial are not uncommon [1,3]. Adverse reactions from SXT may occur in anywhere from 50% to 100% of patients infected with the human-immunodeficiency virus (HIV) who are at high risk of infection by this organism [4]. The worldwide rate of resistance is no more than 10%, although it may be as high as 100% in some regions of the world [1]. In addition, there are obvious discrepancies regarding in vitro activity among potential antimicrobials in different regions and different studies [5]. Thus, determining the most useful drugs to combat this bacterium is of critical importance.

In order to evaluate the efficacy of these drugs, we carried out susceptibility testing against 80 consecutively isolated and 22 SXT-resistant strains of *S*. *maltophilia* with candidates that have been shown in previous studies to demonstrate in vitro antimicrobial activity, including SXT, minocycline, tigecycline, moxifloxacin, levofloxacin, ticarcillin-clavulanate, ceftazidime, polymyxin E, and chloramphenicol [1,3].

Despite a lack of clinical data, treatment of *S*. *maltophilia* with combination antimicrobials is generally recommended for serious infections or when SXT therapy is ineffective [1]. Time-kill curves are considered more relevant in the clinical setting than other methods of combination testing such as checkerboard testing, or etest methods [6,7]. Based on the outcome of susceptibility testing, bactericidal characteristics, and the clinical uses of certain drugs, we selected SXT, minocycline, tigecycline, moxifloxacin and ceftazidime alone and in combinations against 12 clinical isolated strains of *S*. *maltophilia* (including 6 SXT-resistant strains) in time-killing experiments. Given that some previous time-killing studies using drug concentrations based on Minimal Inhibitory Concentration (MIC) or peak levels in serum may not be achievable in the clinical setting [8,9], we carried out our experiments using clinically achievable antimicrobial concentrations.

## Materials and Methods

### Microorganisms and susceptibility testing

The 80 clinical consecutively isolated *S*. *maltophilia* strains were collected between March and November 2013 and the 22 SXT-resistant strains were isolated between January 2012 and October 2013 at our hospital. All strains were identified using the Vitek II system standard and biochemical methods. Of the consecutively isolated strains, 63 (78.8%) were recovered from respiratory tract specimens, followed by 10 (12.5%) from catheter-related specimens, 4 (0.05%) from urinary specimens, and 3 (0.04%) from blood specimens. In contrast, in the 22 SXT-resistant isolates, 17 (77.3%) were from respiratory tract specimens and 5 (22.7%) were from urinary specimens. Antimicrobial susceptibility testing for SXT, minocycline, tigecycline, moxifloxacin, levofloxacin, ticarcillin-clavulanate, ceftazidime, polymyxin E, and chloramphenicol was performed by agar dilution according to the Clinical and Laboratory Standards Institute (CLSI) guidelines [10]. Escherichia coli ATCC 25922 and Pseudomonas aeruginosa ATCC 27853 were chosen as quality control strains in each batch of tests. SXT, minocycline, levofloxacin, ticarcillin-clavulanate, ceftazidime, and chloramphenicol standards were purchased from the National Institute for the Control of Pharmaceutical and Biological Products (NICPBP, Beijing, China). Tigecycline and moxifloxacin were obtained from Wyeth Pharmaceutical (Wyeth Pharmaceutical, Philadelphia, PA, USA) and Bayer Healthcare (Bayer Pharma, Wuppertal, AG, Germany), respectively. Antibiotic solutions except for unstable ones were prepared two weeks in advance and stored at -80°C. The unstable tigecycline ticarcillin-clavulanate and ceftazidime were freshly prepared on the day of use. Mueller-Hinton agar was purchased from Becton, Dickinson and Co. (Difco, Franklin Lakes, NJ, USA).

### Time-kill experiments

Twelve isolates were chosen in the time-kill assays according to the method described previously, using an overnight inoculum of approximately 10^6^ CFU/ml [6]. Considering susceptibility and in vitro antimicrobial characteristics, we chose SXT, minocycline, tigecycline, moxifloxacin and ceftazidime in our experiments. Antibiotic concentrations used in the study were close to their mean free area under the antibiotic concentration-time curve (AUC) steady-state serum concentrations in health human populations calculated by literature data as earlier study described (based on AUC in serum or plasma over 24 h divided by 24 h) [11]. The pharmacokinetic parameters incorporated in time-kill experiments are showed in Table 1. For SXT, the susceptible concentration 38/2 mg/L was used due to a lack of the other data. The following concentrations were used: 2 mg/L for minocycline [12], 0.25 mg/L for tigecycline [13], 1.8 mg/L for moxifloxacin [14], and 5.3 mg/L for ceftazidime [15]. Of the twelve strains selected for the time-kill studies, six were resistant to SXT, three were resistant to minocycline, six were resistant to moxifloxacin and ten were resistant to ceftazidime. Details are shown in Table 2. SXT plus moxifloxacin, SXT plus minocycline, SXT plus tigecycline, minocycline plus moxifloxacin, minocycline plus ceftazidime, tigecycline plus moxifloxacin, and tigecycline plus ceftazidime were chosen in this study. Samples were taken from the liquid cultures at 0, 3, 6, 12, and 24 h, serially diluted, spread on plates, and incubated at 35°C. Bacterial colonies were counted after 24 h. Bacteriostatic activity was defined as a < 3 log_10_ decrease in bacterial concentration compared to that of the initial inocula. Bactericidal activity was defined as a ≥ 3 log_10_ decrease in bacterial concentration compared with the initial inocula. Synergism was defined as a 100-fold increase in killing at 24 h (as measured by colony counts) with the combination, in comparison with the most active single drug. Antagonism was defined as a 100-fold decrease in killing at 24 h with the combination, compared with the most active single drug alone [6].

**Table 1:** Summary of Pharmacokinetic parameters incorporated in time-kill experiments.

Antibiotic	regimen	Unbound fraction(%)	AUC(μg*hr/ ml)	reference
minocycline	200mg po	24	48.3	12
tigecycline	50mg q 12h	20	3.07^a^	12,13
moxifloxacin	400mg q 24h	60	47.97	12,14
ceftazidime	1000mg IV	90	127	12,15

a: 0-12h AUC at steady state for multiple-dose studies.

**Table 2:** *S*. *maltophilia* MIC summary data in the time-killing assays.

No.of isolates	MIC(μg/ ml)				
	SXT	MIN	TGC	MOX	CAZ
1	19/1	2	8	16	>64
2	9.5/0.5	8	8	>16	>64
3	4.75/0.25	0.25	1	0.125	4
4	4.75/0.25	0.5	0.125	1	>64
5	4.75/0.25	4	8	4	4
6	38/4	4	8	32	32
7	76/4	0.5	1	0.25	64
8	≥152/8	16	8	2	64
9	≥152/8	16	2	16	>64
10	≥152/8	0.5	1	8	>64
11	≥152/8	2	1	4	>64
12	≥152/8	4	8	16	>64

SXT: trimethoprim/sulfamethoxazole, MIN: Minocycline, TGC: Tigecycline, MOX: Moxifloxacin, CAZ: Ceftazidime.

## Results

### Susceptibility testing

Susceptibility testing outcomes for 80 consecutively isolated and 22 SXT-resistant strains are shown in Table 3 and Table 4, including MIC_50_ and MIC_90_ (MIC at which 50% and 90% of isolates were inhibited), % resistant, % intermediate, and % susceptible. Given no certain interpretive breakpoints for tigecycline, moxifloxacin, and colistin, we applied the breakpoint for Enterobacteriaceae (susceptibility at ≤ 2 μg ml^-1^, intermediate at 4 μg ml^-1^, and resistance at ≥ 8 μg ml^-1^). Results showed that the preliminary active agents were minocycline and SXT (susceptible against >90% of the consecutively isolated strains) and the non-susceptible agents were ceftazidime, colistin, and chloramphenicol (susceptible against <30% of the consecutively isolated strains). It is noteworthy that except for minocycline and tigecycline, which maintained good susceptibility rates (against >70% of the strains), susceptibility rates were markedly decreased in the 22 SXT-resistant strains for the remaining antimicrobial agents.

**Table 3:** In vitro susceptibility of 80 clinical consecutively isolated strains of *S*. *maltophilia* to nine antibiotics.

MIC(μg/ml)	SXT	MIN	TGC	MOX	LVX	TIM	CHL	CAZ	COL
**MIC**_**50**_	**4.75/0.25**	**0.5**	**1**	**1**	**1**	**4**	**16**	**64**	**16**
**MIC**_**90**_	**9.5/0.5**	**2**	**4**	**8**	**16**	**128**	**32**	**128**	**64**
**S**	**93.8%**	**95.0%**	**83.8%**	**80.0%**	**76.3%**	**76.3%**	**37.5%**	**20.0%**	**22.5%**
**I**	**-**	**1.3%**	**8.8%**	**1.3%**	**5.0%**	**11.3%**	**28.8%**	**12.5%**	**15.0%**
**R**	**6.3%**	**3.8%**	**7.5%**	**18.8%**	**18.8%**	**12.5%**	**33.8%**	**67.5%**	**62.5%**

SXT: trimethoprim/sulfamethoxazole, MIN: Minocycline, TGC: Tigecycline, MOX: Moxifloxacin, LVX: Levofloxacin, TIM: Ticarcillin-clavulanate, CHL: Chloramphenicol, CAZ: Ceftazidime, COL: colistin.

**Table 4:** In vitro susceptibility of 22 SXT -resistant strains of *S*. *maltophilia* to nine antibiotics.

MIC(μg/ml)	SXT	MIN	TGC	MOX	LVX	TIM	CHL	CAZ	COL
**MIC**_**50**_	**152**	**2**	**1**	**4**	**4**	**16**	**128**	**128**	**16**
**MIC**_**90**_	**152**	**16**	**8**	**16**	**32**	**256**	**128**	**128**	**64**
**S**	**0**	**86.4%**	**72.7%**	**40.9%**	**40.9%**	**50.0%**	**13.6%**	**4.5%**	**22.7%**
**I**	**-**	**0**	**9.1%**	**9.1%**	**4.5%**	**22.7%**	**4.5%**	**9.1%**	**9.1%**
**R**	**100%**	**13.6%**	**18.2%**	**50.0%**	**54.5%**	**27.3%**	**81.8%**	**86.4%**	**68.2%**

SXT: trimethoprim/sulfamethoxazole, MIN: Minocycline, TGC: Tigecycline, MOX: Moxifloxacin, LVX: Levofloxacin, TIM: Ticarcillin-clavulanate, CHL: Chloramphenicol, CAZ: Ceftazidime, COL: colistin.

### Time-kill experiments

**Fig 1:**
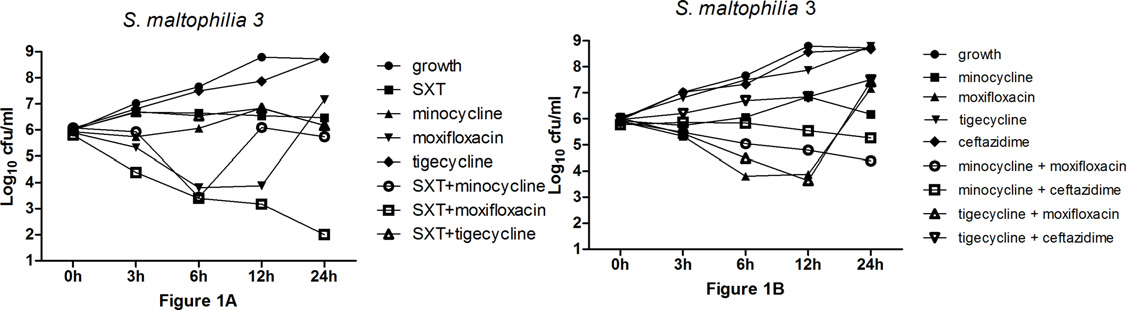
Time-kill curves were generated for *S*. *maltophilia* clinical isolate no. 3.

**Fig 2:**
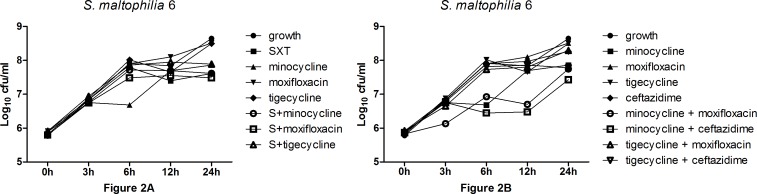
Time-kill curves were generated for *S*. *maltophilia* clinical isolate no.6.

Time-kill curves were generated for representative *S*. *maltophilia* clinical isolate no. 3 and no. 6 (Fig 1 and Fig 2). SXT plus moxifloxacin demonstrated synergy for strain numbers 3, 4, 5, 7, 8, and 11 (Table 5). Of note, all of the moxifloxacin MIC values for these strains were ≤ 4 μg/mL (Table 2). Minocycline plus moxifloxacin demonstrated synergy for strain numbers 8 and 10 (Table 5). Tigecycline plus moxifloxacin demonstrated synergy for strain numbers 8 (Table 5). However, SXT plus minocycline, SXT plus tigecycline, minocycline plus ceftazidime, and tigecycline plus ceftazidime demonstrated indifferent (Fig 2, Table 5). There were no antagonisms in these combinations. Changes in bacterial concentrations in log_10_ CFU/mL (Δ) at 24 h compared with that the starting inoculum (0 h) are summarized in Table 5. Except for strains that were susceptible to moxifloxacin, the majority of the single agents exhibited increasing bacterial concentrations at 24 h compared with that of the initial inocula (Table 5). No single drug exhibited bactericidal activity against *S*. *maltophilia*. In the combination study, a combination drug generally exhibited lower bacterial concentrations compared with the single drug on the condition that at least one drug in the combination was susceptible against *S*. *maltophilia* (Table 2 and Table 5). The combinations including moxifloxacin exhibited bactericidal activity against *S*. *maltophilia* when moxifloxacin was susceptible to this bacterium (Table 5).

**Table 5:** Change in bacterial concentrations in log10 CFU/ml (Δ) at 24 h compared with the starting inoculum (0 h) is shown^a^.

	Δconcentration (log_10_ CFU/ml) of 24 hour^b^											
No.	SXT	MIN	TGC	MOX	CAZ	SXT+MIN	SXT+MOX	SXT+TGC	MIN+MOX	MIN+CAZ	TGC+MOX	TGC+CAZ
1	1.49	1.53	2.73	2.54	2.20	0.95	0.84	1.59	1.63	1.75	3.26	2.96
2	1.02	1.00	3.09	2.26	2.27	0.99	1.39	0.87	1.16	1.17	2.17	1.70
3	1.55	0.08	2.57	-2.03	2.23	0.02	-4.23	1.03	-3.39	-0.05	-2.96	0.91
4	0.46	0.24	2.72	-0.67	2.72	-0.34	-3.82	0.16	-1.51	-0.50	1.41	1.53
5	1.43	1.62	2.66	1.36	2.77	1.15	-1.04	1.06	0.62	0.56	0.96	2.34
6	1.83	1.97	2.68	2.60	2.26	1.83	1.68	1.98	1.92	1.56	2.40	1.91
7	2.15	0.26	2.62	-0.94	2.05	0.56	-3.01	1.21	-1.62	-0.84	-2.18	1.98
8	2.82	2.50	2.37	-0.01	2.00	2.14	-3.30	2.22	-3.97	-0.02	-3.18	1.87
9	3.55	2.60	2.71	2.66	3.14	2.84	2.42	2.89	2.49	2.62	3.47	2.44
10	3.95	-0.07	3.77	3.51	3.63	2.33	0.18	3.78	-2.32	1.50	2.34	3.13
11	2.69	2.60	2.65	2.68	2.07	2.88	-1.86	2.67	1.27	0.10	2.40	2.25
12	3.01	2.48	2.57	2.16	2.39	3.14	1.87	3.09	2.22	2.95	2.42	1.49

SXT: trimethoprim/sulfamethoxazole; MIN: minocycline; TGC: tigecycline; MOX: moxifloxacin; CAZ: ceftazidime.

+:and;-: bacterial concentrations decrease;≥-3: bactericidal activity.

a:time-kill experiments were duplicated; mean values are utilized. In all duplicate experiments, similar results were obtained

b:If the differential of ratio value of combination compared with most effective single drug≥2,we think they are synergy.

## Discussions

In susceptibility testing, SXT and minocycline demonstrated the highest in vitro activity against clinical consecutively isolated strains of *S*. *maltophilia*. Based on high in vitro susceptibility rates, SXT is generally recommended and used in infections caused by *S*. *maltophilia*. However, resistance to this drug has been increasing over the years [1,3,5]. In addition, adverse reactions are not uncommon, especially in HIV-infected patients who are at high risk of infection by this organism [4]. When SXT is contraindicated, determining the appropriate drug is difficult. For infections among the general population, our study demonstrated that minocycline, tigecycline, moxifloxacin, levofloxacin, and ticarcillin-clavulanate might serve as secondary choices. However, if *S*. *maltophilia* is resistant to SXT, moxifloxacin, levofloxacin, and ticarcillin-clavulanate displayed sharp declines in susceptibility against this bacterium (Table 4). The same resistance mechanism, such as multidrug efflux pumps, may apply to these drugs as it does to SXT [16]. In these candidate antimicrobials, minocycline and tigecycline demonstrated good in vitro activity against SXT–resistant *S*. *maltophilia*. Of note, minocycline displayed consistent activity against both SXT-susceptible and SXT-resistant strains. Several published studies have shown that minocycline is not inferior to SXT (and might even be better than SXT) in susceptibility [1,3,5]. Case reports and our previous in vitro Monte Carlo simulation have provided some evidence of its clinical efficacy [17,18]. These results suggest that minocycline may be considered as first-line therapy alongside SXT in *S*. *maltophilia* infections, and even in SXT-resistant strains. Though tigecycline is derived from minocycline and exhibits better susceptibility in other organisms [19], it was inferior to minocycline in both the present study and in our earlier Monte Carlo simulation analysis [18]. Additionally, chloramphenicol, polymyxin E, and ceftazidime demonstrated high levels of resistance against *S*. *maltophilia*. Though susceptibility and combination studies have been widely reported from around the world, the efficacy of SXT, minocycline, tigecycline, moxifloxacin, and ceftazidime alone and in combinations are still unclear as therapeutic strategies [1,3,5].

In the time-kill study, the effectiveness of five antimicrobial agents alone and in combinations were tested against SXT–resistant, minocycline–resistant, tigecycline–resistant, moxifloxacin–resistant, and ceftazidime–resistant clinical isolated strains of *S*. *maltophilia*. In single-agent studies, none of the antimicrobials had bactericidal activity against *S*. *maltophilia* even though moxifloxacin and ceftazidime are usually categorized as bactericidal antimicrobials [20]. Notably, SXT and minocycline exhibited bacteriostatic activity in isolates with susceptible MIC values; bacterial concentrations of these two drugs kept a steady level at 24-hour intervals (Fig 1A). Of note, moxifloxacin exhibited a significant decrease in Colony Forming Units (CFU) at earlier 6 h for strains with susceptible MIC values, then a regrowth at a later time, as shown in Fig 1. This result is similar to that of the study by Evangelos *et al*.; however, their studies were based on four times the MIC for most drug concentrations, which is unachievable in the clinical setting [8]. Overall, we found that except tigecycline and ceftazidime the susceptible isolates (against one certain antimicrobial agent) generally showed lower bacterial concentrations than the resistant ones (Table 5). Tigecycline and ceftazidime exhibited limited activity against *S*. *maltophilia* even in strains that were susceptible to them. Low serum concentrations may explain the limited efficacy of tigecycline, since this drug is usually not recommended for blood stream infections [13]. The high levels of resistance may explain the poor efficacy of ceftazidime, and the Expert Rules in Antimicrobial Susceptibility Testing of the European Committee on Antimicrobial Susceptibility Testing considers *S*. *maltophilia* to be intrinsically resistant to ceftazidime [1]. Except for the strains that were susceptible to moxifloxacin, single drugs all exhibited increasing bacterial concentrations compared with initial inoculum (Table 5). These results demonstrate that single agents may have limited efficacy in infections caused by *S*. *maltophilia*, especially in case of immunocompromised patients.

Since monotherapy is often inadequate, combination therapy has been recommended for synergy and the potential to overcome resistance [21–23]. In contrast to the single-agent experiments, though most of the combinations lack of benefit of synergy, combination regimens displayed better activities inhibiting or killing *S*. *maltophilia*. The bacterial concentrations in combination regimens generally decreased compared with the single-agent experiments (Table 5). Notably, SXT plus moxifloxacin exhibited a consistent killing effect and displayed synergy against all of the six strains with low moxifloxacin MIC values. Moxifloxacin plus minocycline and Moxifloxacin plus tigecycline displayed synergy in few strains. These outcomes suggest that SXT plus moxifloxacin combination may kill the bacteria more rapidly than others or single agents. However, drug combinations were not found to be any more effective than single drugs in bacterial concentrations against *S*. *maltophilia* strains that were resistant to both drugs used in the combinations.

Our study had several limitations. First, all strains were collected at one center and their numbers were relatively small. Therefore, further studies with a greater number of strains from diverse sources are needed to confirm these findings. Second, we used mean steady-state concentrations of non-protein-bound drugs in humans in the time-kill study. Drug concentrations may vary in different organs and across different populations, thus different outcomes may occur accordingly. Finally, the combinations in the present study were used without taking into account the potential adverse reactions caused by their use in the clinical setting.

In summary, our susceptibility testing results suggest that SXT and minocycline may be considered as first-line therapeutic choices in *S*. *maltophilia* infections; tigecycline, moxifloxacin, levofloxacin, and ticarcillin-clavulanate may serve in the second-line setting; and ceftazidime, colistin, and chloramphenicol do not appear to be appropriate choices in any setting. However, monotherapy is inadequate in infection management, especially in case of immunocompromised patients. Combination therapy, especially SXT plus moxifloxacin, may benefit than monotherapy in inhibiting or killing *S*. *maltophilia*. Further studies in both the animal model and the clinical setting are needed to confirm these findings.

## Supporting Information

S1 Table The data for Fig 1 and Fig 2. Every of the data in the table stands for the mean value of two times count of the bacteria.
(XLSX)

## References

[1] Looney W, Narita M, Muhlemann K. *Stenotrophomonas maltophilia*: an emerging opportunist human pathogen. Lancet Infect Dis. 2009; 9: 312–323. 10.1016/S1473-3099(09)70083-0 19393961

[2] Sader HS, Farrell DJ, Flamm RK, Jones RN. Antimicrobial susceptibility of Gram-negative organisms isolated from patients hospitalised with pneumonia in US and European hospitals: results from the SENTRY Antimicrobial Surveillance Program, 2009–2012. Int J Antimicrob Agents. 2014;43:328–334. 10.1016/j.ijantimicag.2014.01.007 24630306

[3] Joanna S. Brooke. *Stenotrophomonas maltophilia*: an Emerging Global Opportunistic Pathogen. Clin. Microbiol. Rev. 2012, 25: 2–41. 10.1128/CMR.00019-11 22232370 PMC3255966

[4] Alan C. Jung MD, Douglas S. Paauw MD. Management of Adverse Reactions to Trimethoprim-Sulfamethoxazole in Human Immunodeficiency Virus-Infected Patients. Arch Intern Med. 1994;154:2402–2406. 7979835

[5] Denton M, Kerr KG. Microbiological and clinical aspects of infection caused by *Stenotrophomonas maltophilia*. Clin Microbiol Rev. 1998, 11: 57–80. 9457429 10.1128/cmr.11.1.57 PMC121376

[6] Pillai S. K., Moellering R. C. & Eliopoulos G. M. Antimicrobial Combinations *In* Antibiotics in Laboratory Medicine. 5th edn (ed Lorian V. L.) 365–424 (Lippincott Williams & Wilkins, Philadelphia, 2005).

[7] Ni W, Shao X, Di X, Cui J, Wang R, Liu Y. In vitro synergy of polymyxins with other antibiotics for Acinetobacter baumannii: a systematic review and meta-analysis. Int J Antimicrob Agents. 2015;45:8–18. 10.1016/j.ijantimicag.2014.10.002 25465524

[8] Evangelos J. Giamarellos-Bourboulis, Karnesis Lazaros, Galani Irene, Giamarellou Helen. In Vitro Killing Effect of Moxifloxacin on Clinical Isolates of Stenotrophomonas maltophilia Resistant to Trimethoprim-Sulfamethoxazole. Antimicrob Agents Chemother. 2002; 46: 3997–3999. 12435710 10.1128/AAC.46.12.3997-3999.2002 PMC132774

[9] Poulos C D, Matsumura S O, Willey B M, Low D E, McGeer A. In Vitro Activities of Antimicrobial Combinations against Stenotrophomonas (Xanthomonas) maltophilia. Antimicrob Agents Chemother. 1995; 39: 2220–2223. 8619571 10.1128/aac.39.10.2220 PMC162918

[10] Performance Standards for Antimicrobial Susceptibility Testing; *Twenty-Fifth Informational Supplement*. CLSI document M100-S25. Wayne, PA: Clinical and Laboratory Standards Institute, 2015.

[11] Tängdén T, Hickman RA, Forsberg P, Lagerbäck P, Giske CG, Cars O. Evaluation of double- and triple-antibiotic combinations for VIM- and NDM-producing *Klebsiella* pneumoniae by in vitro time-kill experiments. Antimicrob Agents Chemother. 2014;58:1757–1762. 10.1128/AAC.00741-13 24395223 PMC3957864

[12] Jay P. Sanford. *The Sanford guide to antimicrobial therapy* 43^rd^ edition.p.86

[13] Meagher AK, Ambrose PG, Grasela TH, Ellis-Grosse EJ. Pharmacokinetic/pharmacodynamic profile for tigecycline-a new glycylcycline antimicrobial agent. Diagn Microbiol Infect Dis. 2005;52:165–171. 16105560 10.1016/j.diagmicrobio.2005.05.006

[14] Rodvold KA, Neuhauser M. Pharmacokinetics and pharmacodynamics of fluoroquinolones. Pharmacotherapy. 2001;21:233S–252S. 11642690 10.1592/phco.21.16.233s.33992

[15] Leroy A, Leguy F, Borsa F, Spencer GR, Fillastre JP, Humbert G. Pharmacokinetics of ceftazidime in normal and uremic subjects. Antimicrob Agents Chemother. 1984;25:638–642. 6375562 10.1128/aac.25.5.638 PMC185604

[16] Sánchez MB, Martínez JL. The efflux pump SmeDEF contributes to trimethoprim-sulfamethoxazole resistance in *Stenotrophomonas maltophilia*. Antimicrob Agents Chemother. 2015;59:4347–4348. 10.1128/AAC.00714-15 25918144 PMC4468665

[17] Falagas ME, Valkimadi PE, Huang YT, Matthaiou DK, Hsueh PR. Therapeutic options for *Stenotrophomonas maltophilia* infections beyond co-trimoxazole: a systematic review. J Antimicrob Chemother. 2008;62: 889–894. 10.1093/jac/dkn301 18662945

[18] Wei Chuanqi, Ni Wentao, Cai Xuejiu, Cui Junchang. A Monte-Carlo pharmacokinetic/pharmacodynamic simulation to evaluate the efficacy of minocycline, tigecycline, moxifloxacin and levofloxacin in the treatment of hospital-acquired pneumonia caused by *Stenotrophomonas maltophilia*. Infect Dis. 2015;47:846–51. 10.3109/23744235.2015.1064542 26167850

[19] Noskin GA. Tigecycline: a new glycylcycline for treatment of serious infections. Clin Infect Dis. 2005;41:S303–314. 16080069 10.1086/431672

[20] Levison ME, Levison JH. Pharmacokinetics and pharmacodynamics of antibacterial agents. Infect Dis Clin North Am. 2009; 23: 791–815. 10.1016/j.idc.2009.06.008 19909885 PMC3675903

[21] Wang YL, Scipione MR, Dubrovskaya Y, Papadopoulos J. Monotherapy with fluoroquinolone or trimethoprim-sulfamethoxazole for treatment of *Stenotrophomonas maltophilia* infections. Antimicrob Agents Chemother. 2014;58:176–182. 10.1128/AAC.01324-13 24145530 PMC3910778

[22] Hand E, Davis H, Kim T, Duhon B. Monotherapy with minocycline or trimethoprim/sulfamethoxazole for treatment of *Stenotrophomonas maltophilia* infections. J Antimicrob Chemother. 2016 1 21. [Epub ahead of print] 10.1093/jac/dkv456 26801080

[23] Zelenitsky SA, Iacovides H, Ariano RE, Harding GKM. Antibiotic combinations significantly more active than monotherapy in an in vitro infection model of *Stenotrophomonas maltophilia*. Diagn Microbiol Infect Dis.2005; 51:39–43. 15629227 10.1016/j.diagmicrobio.2004.09.002

